# Cooperative Team Learning and the Development of Social Skills in Higher Education: The Variables Involved

**DOI:** 10.3389/fpsyg.2018.01536

**Published:** 2018-08-22

**Authors:** Santiago Mendo-Lázaro, Benito León-del-Barco, Elena Felipe-Castaño, María-Isabel Polo-del-Río, Damián Iglesias-Gallego

**Affiliations:** Department of Psychology and Anthropology, Faculty of Teacher Training, University of Extremadura, Badajoz, Spain

**Keywords:** social skills, cooperative learning, teamwork, quasi-experimental research, university students

## Abstract

The cooperative methodology provides an opportunity for university students to develop interpersonal, social, and teamwork competences which can be decisive in their professional and social success. The research described here examines the influence of cooperative learning on the social skills necessary for teamwork. Furthermore, it analyses whether the continued use of this type of learning, the type of group, the basic social skills for teamwork, or the academic level of the students, influence their efficacy. To do so, we have designed a research project of a quasi-experimental kind with a pre-test, a post-test, and a control group, in which 346 university undergraduate students studying degrees in Infant and Primary Education completed self-report surveys about behavior patterns in social skills concerning self-assertion and the reception and imparting of information in teamwork situations. The results show that cooperative learning in university classrooms is effective as a method for developing the social skills necessary for teamwork, as well as the relevance of the control over the number of students in a group, the basic social skills, or the academic level of the students, as relevant factors related with efficacy; where continuity over time in the use of the cooperative methodology is what marks the greatest differences in the development of the social skills necessary for teamwork. It is important to stress that when students are asked to work autonomously in teams, with the aim of favoring the development of social skills, they should be given adequate structures that can guarantee the minimum conditions of participation, so as to allow a proper development of the said social skills.

## Introduction

There is a growing impetus toward cooperation and the encouragement of participation in all kinds of educational, labor, and social organizations, where the individual is being replaced as the main productive unit by a great variety of teams and workgroups (Eurofound, 2007; Gil and Alcover, 2008); it is, therefore, a competence increasingly in demand in the labor market. To be precise, collaborative social skills are becoming ever more valuable and valued by employers (OECD, 2015).

Similarly, cooperative or collaborative learning, or other forms of group learning, have been the subject of the utmost interest over the last few decades, for their implications on educational, social, and work levels. Nevertheless, finding ways to organize and conduct classes so as to reconcile the maximum learning with the education of persons who can cooperate and establish good human relationships is a great educational challenge (Goikoetxea and Pascual, 2002; St-Pierre and Richer, 2008).

With the aim of covering these necessities, many teachers turn to group work, so the educational methods based on cooperation have spread rapidly throughout the world, becoming more and more common in the classroom, to promote teamwork among the students, learning to work in teams, improving performance and learning or developing interpersonal competences (Manzer and Bialik, 1997; Venter and Blignaut, 1998; Boling and Robinson, 1999; Gottschall and García-Bayonas, 2008; Jones and Jones, 2008; Gaudet et al., 2010; Mendo et al., 2016).

One of the methodologies most used by teachers for grouping students is cooperative learning. For a long time, cooperative methods have been very popular at primary and secondary school levels, so the great majority of studies concerning cooperative learning have been with children. However, although academic interest in studying cooperative learning in higher education has recently increased, empirical evidence of its impact at university level is still limited (Herrmann, 2013).

It is very important to prepare students to cooperate (Blatchford et al., 2003; Johnson and Johnson, 2006; Webb, 2009). In most cases, students are not accustomed to working cooperatively, so the interactions may not develop in the desired way, even when they have cooperative instructions (Buchs and Butera, 2015).

The university lecturer should create the necessary conditions to guarantee the efficacy of the learning teams. Achieving the multiple aims of cooperative learning in the university classroom requires careful planning on the teacher’s part, along with interventions throughout the process to resolve conflicts and a post-analysis of the teamwork (León et al., 2015).

### Development of Social Skills Through Cooperative Learning

Social skills are behaviors through which we express ideas, feelings, opinions, affection, maintain or improve our relationship with others, and solve and strengthen a social situation (León, 2009).

Having cooperative learning in the classroom implies learning together in groups, while also acquiring social skills for working in a team, as the cooperative environment is ideal for developing the adequate social skills (Jordan and Métais, 1997; Rutherford et al., 1998; Lavasani et al., 2011; Turrión and Ovejero, 2013).

The group members learn by bringing into play a series of essential social skills what fully engage social cognition, insofar as they require understanding social habits and norms that lead to cooperation between the team members (Brizio et al., 2015).

There are important similarities between cooperative learning and training in social skills. In cooperative learning, the students imitate other companions (modeling), practice the learnt communicative and social skills (behavioral trials), and receive information immediately concerning their behavior from their companions (feedback). Finally, they transfer what they have learnt to other, different situations (generalization) (León et al., 2015). In short, the cooperative learning group would function as a training group in social skills.

Although the theoretical suppositions underlying both techniques (cooperative learning and training in social skills) are to a great extent the same (Curran, 1985), the interaction between them favors the acquisition and development of the latter (Echeita and Martín, 1990; Buchs and Butera, 2015; Casal, 2016; Larraz et al., 2017; Vallet et al., 2017).

Cooperative learning is itself an environment in which social skills are acquired or improved; this is mainly due to the key role played by social interaction in the development, not only of academic intelligence, but also social intelligence. So, favoring social interaction between persons contributes to their acquisition of a better socio-cognitive understanding, and this in turn helps to develop social interaction of a better quality (Lacasa, 1993).

### Variables Involved in Effective Cooperative Learning

Cooperative learning has been the object of numerous works of research since the 1970s concerning the academic, social, and affective aspects. Yet, there have been few works of research aimed at solving questions concerning the mediating mechanisms involved in the efficacy of the cooperative methodology (León et al., 2014).

Although the variables that condition the efficacy of cooperative learning are very numerous, when the aim is to develop social skills through teamwork, some authors consider that those related with planning the cooperative learning have particular relevance; such variables would include: the time taken, student groupings, their personal characteristics, the basic social skills, or the students’ academic level.

So, Johnson et al. (1990), Echeita (1995), or Lobato (1997), for instance, state that the existence of social skills among group members determines the efficacy and results of the cooperative learning methodology.

However, the necessary social skills for effective cooperative work do not simply appear when working with this methodology; it is necessary for them to be purposely taught as academic skills *per se* (Gillies and Haynes, 2011). Those students who have never worked under such conditions lack the social skills needed to adequately complete the activity (Domingo, 2010). Thus, in order to implement cooperative learning in the classroom, it is necessary to first, explicitly and systematically, teach how to work in a team, as well as to develop and/or reinforce their existing cooperative social skills (Prenda, 2011).

Nevertheless, as they work together cooperatively, then the basic social skills as a whole will gradually come to dominate in the group (Casey et al., 2009; Lavasani et al., 2011). Thus, the team members will practice what Johnson and Johnson (1991) called social relationship skills.

On the other hand, the interactive process with their colleagues is complex and is determined by the different student groupings established by the teacher.

There are basically three different ways of grouping students that the teacher can choose when planning cooperative learning: formal groups (which may last from a single class to various weeks), informal groups (for a particular activity), and base groups (for a semester or academic year). These three types are different in their implementation as well as in the results of both academic and social learning (Johnson and Johnson, 2014). They usually have between two and four members, although the actual number will depend on the objectives, age and experience of the students, the materials and equipment to be used, and the time (Johnson et al., 1999).

According to Ferreiro (2007), putting students into formal groups has certain benefits, in particular, the development of social skills among the members. To be precise, putting students into pairs or groups strengthens their social skills, as it helps them to become more tolerant toward their companions, to respect them and to pay them more attention (Sharan, 2014). Similarly, maintaining the groups with the same members over time develops feelings of belonging and social skills (Fernández-Rio and Méndez-Giménez, 2016). For Johnson et al. (1999), those groups that stay together for at least a year with the same members, and whose principal objective is to help each other and give mutual support, enable a good cognitive and social development.

Other works of research show the influence of cooperative learning on the students’ academic performance, with greater benefits if the class group is first trained in social skills (Slavin, 1990; Johnson and Johnson, 1999; León, 2006; Kagan and Kagan, 2009; Turrión and Ovejero, 2013).

Although there is evidence of the existing relationship between academic performance and social skills (Lewis, 2007), in a cooperative learning environment in the university, Cadoche (2007) did not find a clear relation. However, he did find that the students with the lowest academic performance had difficulties to communicate and to resolve conflicts; while those with an average performance possessed greater possibilities of growth in social competences, and those who had a high level of academic achievement possessed interactive skills and greater possibilities of success in the social sphere. Along these lines, Neber et al. (2001) specifically confirmed the positive effect of cooperative learning in students who had high performance levels, since it favors, among other things, the development of their social skills.

In one way or another, for team work to progress through cooperative learning, it is necessary for the students to have adequate levels of social skills. The question is whether this methodology can, of itself, be effective in helping the students to acquire, or to increase, their social skills for working in groups and which other variables can be mediating in this process.

### The Present Study

We start from the idea that the cooperative methodology was conceived to enable the development of essential competences for university students in general and future teachers in particular. So, in this study, our aim is to prove that the cooperative methodology in the university context is effective for improving the social skills necessary for team work. On the other hand, as part of a wider study within the framework of research related to the factors that mediate in the effectiveness of learning, in relation to the social skills needed for working in learning groups, we analyze whether the duration of the intervention, the type of grouping (formal, informal, or number of members), the basic social skills for working in teams, or the students’ academic level, influence the efficacy of the cooperative methodology.

## Materials and Methods

### Participants

There were 346 students (78% women) from the Degrees in Infant Education (IE) (*n* = 154, 44.5%) and Primary Education (PE) (*n* = 192, 55.5%) at the University of Extremadura (Spain) participating in this research. They were between 18 and 36 years of age, the mean age being 20.52 (*SD* = 2.48) years. The selection criterion for the experimental group (*n* = 220, IE = 104; PE = 116) was being registered to study at least one subject during the 2015–2016 academic year in which at least one of the lecturers had specific training in cooperative learning. As a control group (*n* = 126; IE = 70; PE = 56), in order to have the greatest possible equivalence between groups, students were chosen from the same degree courses, but from subjects whose lecturers did not use cooperative learning in the classroom. There were no significant differences between the experimental and control groups as far as age, *t*(344) = 1.397, *p* = 0.163, gender, χ^2^(1) = 1.361, *p* = 0.243, or academic level, *t*(344) = −1.597, *p* = 0.111, were concerned.

We believe it should be pointed out that, in the academic guides to the Degrees in question, there is a large quantity of information and activities relating to group work, as well as the academic and social competences related to the said contents and activities. So, for the students who were participating in the study, group work is a very important part of their formation.

### Instruments and Variables

#### Questionnaire on the Social Skills of Teamwork Learning (CHSEA)

This questionnaire evaluates the social skills that the students manifest when working in learning groups in a university environment. It is made up of 15 items that are answered using a 5-point Likert scale (from 1 = “Totally disagree” to 5 = “Totally agree”). The CHSEA evaluates three factors of the social skills needed for team learning: (1) self-assertion: messages in the first person singular, asking for changes in behavior, taking criticism on board, stopping interaction (e.g., “I express my opinions and feelings appropriately”); (2) receiving information: actively listening, empathizing, summarizing, asking for help, asking questions (e.g., “I actively listen to the contributions of others”); (3) imparting information: motivating, imparting information, convincing others, explaining oneself, giving help (e.g., “I provide valuable information to my classmates”). A higher score in each of the factors, or in the total score, is an indicator of greater social skills.

The Alpha indices (α = 0.84) and Compound Reliability (CR = 0.98) indicate a good global reliability of the CHSEA, with a average variance extracted (AVE = 0.63). The three factors of the questionnaire present an adequate reliability and AVE ≥ 0.50, F1 (α = 0.77, CR = 0.81; AVE = 0.50); F2 (α = 0.80, CR = 0.86, AVE = 0.55); F3 (α = 0.79, CR = 0.86, AVE = 0.56).

#### Grouping the Students

The students were grouped in teams of four and two students. Of these groups, half always worked with the same group or partner (formal groups) and the other half changed teams or partner (informal groups).

#### Time

In addition, approximately 50% of the students, working in formal or informal teams, in groups or pairs, worked cooperatively over one semester, while the rest did so for the whole academic year. Thus, the number of students was equivalent with respect to the number of members in the group, the type of grouping and the duration of the intervention.

#### Basic Social Skills

In order to check whether the increase in the CHSEA scores after training is dependent upon the social skills with which the students began (Basic social skills), using a percentile criterion, the total CHSEA score in the pre-test was divided into three groups of equal size (33%), assuming that the lower, medium and upper thirds of the sample correspond to those with low, medium, and high social skills, respectively.

#### Academic Level

The academic level of the students was evaluated using the mean score of their university academic record. On this basis, three groups of equal size (33%) were established, assuming that the lower, medium, and upper thirds of the sample correspond to the low, medium, and high academic levels, respectively.

### Design

A quasi-experimental methodology of an inter-group pre-test/post-test design with group control has been used, in which the participation of the subjects is not random, since the groups are made up naturally and cannot be formed in a random manner (Campbell and Stanley, 2015). This is because the aim is to maintain the reality of the classroom and the conditions proper to it. A quasi-experimental design means that experimental designs are applied to real situations (educational, family, social, etc.). The two fundamental strategies used to mitigate the defects of this methodology are: (1) the inclusion of a control group; and (2) taking measurements before and after the application of the treatment.

The experimental group is made up of four sub-groups (first and second years of the Degrees in Infant and Primary Education). Their experimental condition is the application of cooperative learning techniques in the classroom, although the experimental condition differs as far as the time of intervention and form of grouping are concerned, since: (1) the students in IE (*n* = 105) worked cooperatively in two subjects, one each semester; (2) those in PE (*n* = 115) worked cooperatively in one subject in the second semester; (3) the first year students (*n* = 112) always worked in the same group (*n* = 60) or pair (*n* = 52); while (4) the second year students (*n* = 108) worked with different groups (*n* = 56) or pairs (*n* = 52). We aimed to achieve the best possible heterogeneity in the formation of the cooperative work teams as far as gender, age, and academic level were concerned.

So that all the experimental groups should start from the same comparative basis, an agreement was reached to use eight times per subject of 1.5 h duration (a total of 12 h) cooperative techniques to learn, organization, consolidation, and discrimination of concepts, that would require a high level of cooperation among the group members, and allow greater flexibility, when adapting to the needs of numerous classroom groups (e.g., “Jigsaw, Cooperative Tables, Cooperatives Maps, Cooperative Dyads”; see Slavin, 1990; León et al., 2005; Macpherson, 2015). These cooperative learning techniques guarantee students’ responsibility (putting out maximum effort) and interdependence (depending on each other to achieve the goal). As well as the interaction between students, that favors the acquisition and development of social skills.

The control group received no intervention and was made up of students from two groups, (first year of the Infant Education degree and second year of the Primary Education degree), working in traditional teamwork methods (i.e., the teacher simply told the students to form groups, either at random or according to the students’ wishes, in order to carry out a task, normally working on their own until the task was finished, with no group processing and with the stress falling on the task to be carried out. In this way, the interdependence, individual responsibility, and communication skills are taken for granted or are not taken into account.

### Procedure

The project began with a period of training for the participating teachers on cooperative learning (dynamics and techniques, role of the teacher, and evaluation. We insisted on the importance of regularly observing the group’s interactions and progress, intervening when necessary to help the students advance in the task, providing feedback concerning their performance and the evaluation (both individual and group), as well as to establish space for reflection on what worked well in the group and what could be improved.

As for ethical norms, the study received approval from the Ethics Committee of the University of Extremadura. Prior to administering the questionnaire, following the ethical directives of the American Psychological Association (2009), the students gave their informed consent to participate in the research, guaranteeing the anonymity and confidentiality of the data and their exclusive use for research purposes. All the experimental and control sub-groups underwent pre-intervention and post-intervention evaluations.

### Data Analysis

Through the statistical package SPSS (version 21), were of used the student *t*-tests for related samples and covariance analysis (ANCOVA). Tests to check the size of the effect (Cohen’s *d* and η^2^) and evaluate the reliability of the factorial structure of the CHSEA (α, CR, and AVE) were also used. Data was subjected to the Kolmogorov–Smirnov (*p* > 0.05) and Levene’s tests, were not significant was found. This way, the assumptions of normality and homoscedasticity, were contrasted.

## Results

### Effectiveness of Cooperative Learning in Teamwork Social Skills

First of all, inter-group (control/experimental) pre-test and intra-group (pre-test/post-test) comparisons were carried out (**Table 1**) in order to: (1) know whether the pre-test scores of the CHSEA allow for an appropriate intergroup comparative base (control-experimental) (Hedrick et al., 1993), thus reducing the possibility that the estimates associated with the independent variable, but due to other factors, were not taken into account (Cook and Campbell, 1986); and (2) check whether the work in the classroom using cooperative learning techniques improved the team’s social skills. In addition, to complete the information provided by the application of the significance tests (student *t*), the size of the effect was calculated (**Table 1**) using the *d* statistic proposed by Cohen (1977).

**Table 1:** Differences between intra- and inter-group averages and size of the effect of the CHSEA (control/experimental groups).

Variables	Control group (*n* = 126)							Experimental group (*n* = 220)							Control/experimental		
	Pre-test		Post-test		Intra-group			Pre-test		Post-test		Intra-group			Pre-test inter-group		
	*M*	*SD*	*M*	*SD*	*t*	*p*	*d*	*M*	*SD*	*M*	*SD*	*t*	*p*	*d*	*t*	*p*	*d*
Total CHSEA	59.87	6.80	59.10	7.40	3.450	0.001	−0.14	60.06	6.35	62.45	5.86	−6.491	<0.001	0.50	−0.311	0.756	0.04
F1 CHSEA	18.30	3.13	17.81	3.25	3.605	<0.001	−0.19	18.62	2.89	19.79	2.73	−5.820	<0.001	0.50	−0.743	0.458	0.13
F2 CHSEA	22.09	2.64	22.04	2.84	0.221	0.826	−0.02	21.77	2.46	22.49	2.28	−4.931	<0.001	0.35	1.126	0.261	−0.15
F3 CHSEA	19.48	2.95	19.33	3.24	1.369	0.173	−0.06	19.67	2.90	20.14	2.67	−2.405	0.017	0.20	−0.901	0.368	0.08

*CHSEA = Questionnaire on team learning social skills: CHSEA factors: F1 = Self-assertion skills; F2 = Information reception skills; F3 = Imparting information skills.*

In the inter-group (control/experimental) pre-test comparisons, the lack of any significant differences between both groups in the CHSEA demonstrates the equivalence between the groups as a basis for comparison. Nevertheless, to eliminate possible variations due to differences between the control group and the experimental group, the data were subjected to the Student’s *t-*test for related samples, contrasting whether the results found could be attributed to the independent variable (application of cooperative techniques in the classroom). In this respect, the intra-group comparisons showed significant improvements (*p* < 0.05) between the pre-test and post-test scores within the experimental group, in the total score and those of the three factors of the CHSEA, with medium effect sizes in the total and factor 1 scores (self-assertion) and small effect sizes in factors 2 (information reception) and 3 (imparting information). On the other hand, within the control group, there was a significant reduction (*p* < 0.05) between the pre-test and post-test scores, in the total score and factors 1 (self-assertion skills) and 3 (imparting information skills) of the CHSEA.

### Mediating Factors of the Effect of Cooperative Learning on Social Skills

Furthermore, taking into account the fact that the students in the experimental group of the degree in Infant Education worked cooperatively for twice the time the students in the degree of Primary Education did, it would seem relevant to present the results separately. Thus, comparisons are once more made between inter-group (Degrees in Infant/Primary Education) and intra-group (pre-test/post-test) averages (**Table 2**).

**Table 2:** Differences between intra- and inter-group averages and size of the effect of the CHSEA in the experimental groups that worked cooperatively over one or two semesters.

Variables	Infant school group (*n* = 104) two semesters							Primary school group (*n* = 116) one semester							Infant/Primary		
	Pre-test		Post-test		Intra-group			Pre-test		Post-test		Intra-group			Pre-test inter-group		
	*M*	*SD*	*M*	*SD*	*t*	*p*	*d*	*M*	*SD*	*M*	*SD*	*t*	*p*	*d*	*t*	*p*	*d*
Total CHSEA	59.20	6.50	63.10	6.11	−6.370	<0.001	0.84	60.60	6.22	62.05	5.68	−3.288	0.001	0.33	−1.586	0.114	0.27
F1 CHSEA	18.19	2.86	20.10	3.02	−5.557	<0.001	0.78	18.88	2.88	19.60	2.52	−2.986	0.003	0.34	−1.736	0.084	0.28
F2 CHSEA	21.71	2.21	22.48	2.24	−3.153	0.002	0.40	21.81	2.61	22.49	2.31	−3.778	<0.001	0.35	−0.276	0.782	0.04
F3 CHSEA	19.30	3.07	20.48	2.77	−4.085	<0.001	0.52	19.90	2.78	19.93	2.59	−0.088	0.930	0.01	−1.510	0.132	0.25

*CHSEA = Questionnaire on team learning social skills: CHSEA factors: F1 = Self-assertion skills; F2 = Information reception skills; F3 = Imparting information skills.*

In this sense, no significant differences (*p* < 0.05) can be appreciated in the inter-group pre-test comparisons. However, the intra-group comparisons demonstrate that the students in the degree of Infant Education achieve significant improvements (*p* < 0.05) in all the variables of the CHSEA, with a large size in the effect for the total score and factor 1 (self-assertion skills), medium size in factor 2 (information reception skills), and small size in factor 3 (imparting information skills). As for the group of students in the degree of Primary Education, the results show that they obtain significant improvements (*p* < 0.05) and small size effects (*d* ≤ 0.35) in the total score, and factors 1 (self-assertion skills) and 2 (information reception skills).

In addition, a multivariate analysis of covariance (MANCOVA) was carried out. This was done, on the one hand, to confirm that the improvements in team social skills observed in the experimental group are conditioned by the time cooperative learning used and, on the other, to analyze whether such factors as the type of student grouping, the basic social skills or the students’ academic level mediate in the effectiveness of cooperative learning as a tool for the development of team social skills. The post-test scores of the CHSEA are used in the MANCOVA as dependent variables, while the pre-test scores of the dependent variables are used as co-variables. As fixed factors, we use: (1) Time (Degree in Infant or Primary Education); (2) Grouping (same group/different group and number of students in team); (3) basic social skills (high/medium/low) and (4) academic performance (high, medium, or low).

Multivariate analysis of covariance revealed significant multivariate effects of the time students worked cooperatively in the experimental group [Wilks λ = 0.952, *F*(4,212) = 2.657, *p* = 0.034, η = 0.048].

Likewise, the univariate contrasts (**Table 3**) confirms the results, which can be seen in **Table 2**; showing that the students who worked cooperatively over the entire academic year (Degree in Infant Education) achieved significantly greater (*p* ≤ 0.05) improvements than the students in the Degree in Primary Education (second semester), in the total score and in the factors 1 (self-assertion skills) and 3 (imparting information skills) of the CHSEA.

**Table 3:** Estimated marginal means and tests of between-subjects effects of cooperative work time: experimental group.

Dependent variables	Time	Estimates		Tests of between-subjects effects					
		*M*	*SE*	*df*	Error	Mean square	*F*	*p*	η
Total	Two semesters	63.56	0.50	1	215	164.609	7.759	0.006	0.035
CHSEA	One semester	61.76	0.40						
F1 CHSEA	Two semesters	20.30	0.26	1	215	34.635	5.997	0.015	0.027
	One semester	19.47	0.21						
F2 CHSEA	Two semesters	22.54	0.20	1	215	0.465	0.140	0.709	0.001
	One semester	22.45	0.16						
F3 CHSEA	Two semesters	20.68	0.25	1	215	38.843	7.456	0.007	0.034
	One semester	19.80	0.20						

*CHSEA = Questionnaire on team learning social skills: CHSEA factors: F1 = Self-assertion skills; F2 = Information reception skills; F3 = Imparting information skills.*

In addition, in relation to the grouping of students, MANCOVA revealed significant multivariate effects of the number of team members [Wilks λ = 0.952, *F*(4,210) = 2.651, *p* < 0.034, η = 0.048]. No significant multivariate effects (*p* ≤ 0.05) were found with respect to working cooperatively in the same team (Formal Group) or different teams (Informal Group) [Wilks λ = 0.982, *F*(4,210) = 0.936, *p* = 0.444, η = 0.018], or the interaction between both variables [Wilks λ = 0.974, *F*(4,210) = 1.395, *p* = 0.237, η = 0.026].

The univariate contrasts (**Table 4**), shows the existence of differences with respect to the number of students in the team, in factor 1 (self-assertion skills), while the students who had worked in groups of 4 had improved the most.

**Table 4:** Estimated marginal means and tests of between-subjects effects of the type of grouping: experimental group.

Dependent variables	Grouping	Estimates		Tests of between-subjects effects					
		*M*	*SE*	*df*	Error	Mean square	*F*	*p*	η
Total CHSEA	Same group	62.52	0.51	1	213	28.331	1.289	0.258	0.006
	Different group	62.38	0.41						
	Two students	62.08	0.47	1	213	1.078	0.049	0.825	0.000
	Four students	62.82	0.44						
F1 CHSEA	Same group	19.96	0.26	1	213	33.791	5.866	0.016	0.027
	Different group	19.64	0.21						
	Two students	19.40	0.24	1	213	5.114	0.888	0.347	0.004
	Four students	20.20	0.23						
F2 CHSEA	Same group	22.32	0.20	1	213	0.000	0.000	0.991	0.000
	Different group	22.59	0.16						
	Two students	22.45	0.18	1	213	3.558	1.065	0.303	0.005
	Four students	22.46	0.17						
F3 CHSEA	Same group	20.15	0.25	1	213	1.568	0.289	0.592	0.001
	Different group	20.14	0.21						
	Two students	20.23	0.23	1	213	0.006	0.001	0.975	0.000
	Four students	20.06	0.22						

*CHSEA = Questionnaire on team learning social skills: CHSEA factors: F1 = Self-assertion skills; F2 = Information reception skills; F3 = Imparting information skills.*

Concerning the effect of the students’ basic social skills and academic level, taking into account the results shown in **Table 1**, those students in the experimental group improved their team social skills, while those in the control group showed a decrease in the total score and in F1 (self-assertion skills) of the CHSEA; so it was considered relevant to carry out the MANCOVA in both groups.

Regarding basic social skills, MANCOVA revealed significant multivariate effects of basic social skills in the control group [Wilks λ = 0.918, *F*(8,394) = 2.142, *p* = 0.031, η = 0.042]. No significant multivariate effects were found in the experimental group [Wilks λ = 0.957, *F*(8,422) = 1.162, *p* = 0.321, η = 0.022].

The univariate contrasts showed a significant effect of basic social skills in the control group for the total score of the CHSEA and the factors 1 (self-assertion) and 3 (imparting information) (**Table 5**). The multiple comparisons of Bonferroni indicate the students in the “low” social skills group in compared to those with “high” social skills, show a significantly greater decrease of them, in the total score (*p* = 0.002) and the factor 3 (*p* = 0.001), and in the factor 1, in comparison with the students with “high” (*p* = 0.001) and “medium” (*p* = 0.009) social skills.

**Table 5:** Estimated marginal means and tests of between-subjects effects of basic social skills: control group.

Dependent variables	Basic social skills	Estimates		Tests of between-subjects effects					
		*M*	*SE*	*df*	Error	Mean square	*F*	*p*	η
Total CHSEA	Low	57.71	0.51	2	120	34.543	6.042	0.003	0.091
	Medium	59.71	0.54						
	High	60.13	0.40						
F1 CHSEA	Low	16.80	0.31	2	120	14.735	7.010	0.001	0.105
	Medium	18.46	0.32						
	High	18.37	0.24						
F2 CHSEA	Low	22.70	0.41	2	120	5.943	1.617	0.203	0.026
	Medium	21.42	0.43						
	High	21.86	0.32						
F3 CHSEA	Low	18.67	0.24	2	120	9.547	7.901	0.001	0.116
	Medium	19.53	0.25						
	High	19.91	0.18						

*CHSEA = Questionnaire on team learning social skills: CHSEA factors: F1 = Self-assertion skills; F2 = Information reception skills; F3 = Imparting information skills.*

On the other hand, MANCOVA revealed significant multivariate effects of the academic level of the students in the control group (Wilks λ = 0.714, *F*(8,218) = 5.296, *p* < 0.001, η = 0.155) and experimental group (Wilks λ = 0.918, *F*(8, 382) = 2.142, *p* = 0.031, η = 0.042). The univariate contrasts revealed a significant effect of the students” academic level, on the total score of the CHSEA and on factor 3 (information dissemination skills) in both groups (**Table 6**).

**Table 6:** Estimated marginal means and tests of between-subjects effects of the academic level.

Group	DV	Academic performance	Estimates		Tests of between-subjects effects				
			*M*	*SE*	*df*	Error	Mean square	*F*	*p*	η
Control	Total CHSEA	Low	58.80	0.31	2	112	30.797	5.020	0.008	0.082
		Medium	59.49	0.47						
		High	60.74	0.51						
	F1 CHSEA	Low	17.71	0.20	2	112	5.662	2.372	0.098	0.041
		Medium	17.80	0.29						
		High	18.53	0.32						
	F2 CHSEA	Low	22.36	0.24	2	112	7.479	2.031	0.136	0.035
		Medium	21.50	0.36						
		High	21.86	0.40						
	F3 CHSEA	Low	18.98	0.14	2	112	15.069	12.838	<0. 001	0.186
		Medium	19.79	0.21						
		High	20.24	0.23						
Experimental	Total CHSEA	Low	61.24	0.51	2	214	77.647	3.668	0.027	0.035
		Medium	62.38	0.53						
		High	63.48	0.67						
	F1 CHSEA	Low	19.32	0.27	2	214	13.350	2.216	0.112	0.022
		Medium	20.01	0.29						
		High	20.13	0.36						
	F2 CHSEA	Low	22.42	0.20	2	214	9.443	3.004	0.052	0.029
		Medium	22.04	0.21						
		High	22.84	0.26						
	F3 CHSEA	Low	19.44	0.25	2	214	21.473	4.183	0.017	0.040
		Medium	20.28	0.26						
		High	20.50	0.33						

*DV, Dependent variables; CHSEA = Questionnaire on team learning social skills: CHSEA factors: F1 = Self-assertion skills; F2 = Information reception skills; F3 = Imparting information skills.*

In addition, the multiple comparisons of Bonferroni indicate that the students of the control group with a high academic performance, decrease to a lesser extent in the total CHSEA score (*p* = 0.006), in comparison with the students with a low academic performance, and in factor 3 (imparting information skills) in comparison with the students with a medium academic performance (*p* = 0.004) and low (*p* < 0.001). While the students of the experimental group with a high academic performance, they improve to a greater extent in the total score CHSEA (*p* = 0.025), and in factor 3, (*p* = 0.033), compared to the students with a low academic performance.

## Discussion

On the one hand, as part of a wider study, this work has examined whether the cooperative methodology is effective as a way of improving social skills for working in a team in the university context, and whether such variables as the time the intervention lasts, the type of grouping, the basic social skills, or the academic level influence the effectiveness of the said methodology in relation to the social skills needed for working in learning groups.

In general, the results of this research corroborate the existence of similarities between the cooperative methodology and training in social skills (León et al., 2015).

Bearing in mind that values of *d* ≥ 0.30 are considered relevant in educational contexts (Borg et al., 1993; Valentine and Cooper, 2003), it can be affirmed that the cooperative methodology has proven to be effective, appreciating significant improvements and relevant effect sizes in the CHSEA.

Nevertheless, in previous research lasting one semester, in which classical training in social skills was applied to university students (Mendo et al., 2016), significant improvements were achieved in all the factors of the CHSEA, with effect sizes of *d* ≥ 0.60 in self-assertion and imparting information and *d* > 0.30 in those of information reception. This would indicate that, in the short term, classical training in social skills is more effective.

On the other hand, the differences found in the effect of the cooperative methodology, with respect to the time of use, show the importance of providing a continuity which forces the students to make use of teamwork social skills (Turrión and Ovejero, 2013). Once the cooperative activities have been planned and carried out several times, greater proficiency is acquired and the classroom routines become automatic, improving the effectiveness.

To be more precise, a longer time of use for the cooperative methodology had a notable effect on imparting information (motivating, imparting information, convincing the others, explaining oneself or helping) and a medium effect on self-assertion (messages in the first person, asking for changes in behavior or receiving criticism). As for receiving information (actively listening, empathizing, summarizing, asking for help or asking questions), the effect of temporal continuity is almost non-existent. This result would suggest, at least in appearance, that the smaller effect on the development of information reception skills in the cooperative methodology could be due to the ceiling effect, since the mean scores obtained for information reception in the pre-test are very high, thus decreasing the margin for improvement, which in turn makes the effective interpretation of the impact more difficult.

As for student groupings, and in accordance with the fact that the number of students in the cooperative team should depend on the desired objectives (Johnson et al., 1999), it could be said that, in a structured group work context that allows cooperative learning in the university, when the objective is the development of social skills, then the type of grouping (formal/informal) is not especially relevant. However, the number of students in a group is relevant here, since a larger number of students in a group would favor the development of self-assertion, i.e., the ability to be assertive concerning one’s own ideas, values or desires, as well as with those of others.

Concerning basic social skills as a mediating factor of the effect of cooperative learning on social skills, training programs in social skills look for the participation of already trained subjects, capable of providing assistance and acting as models for those with greater difficulties, although they themselves also benefit (Caballo, 1993); so, independently of the basic social skills, the training results are similar (Mendo et al., 2016). In this sense, the results show that when work is cooperative, starting off with greater or fewer teamwork social skills was not a determining factor for development. This reaffirms the similarities between cooperative learning and training in social skills (Curran, 1985; León et al., 2015). On the other hand, and in accordance with the existence of social skills among the group members being a necessary requisite (Domingo, 2010; Gillies and Haynes, 2011; Prenda, 2011) and a determining factor in the results of the cooperative learning methodology (Johnson et al., 1990; Echeita, 1995; Lobato, 1997), this would indicate that most of the university students who participated in the study had at least a minimum of social skills that allowed them to use the said skills in the exchanges with their colleagues (Turrión and Ovejero, 2013). This in turn meant that, as the group worked together, their social skills improved (Casey et al., 2009; Lavasani et al., 2011).

Nevertheless, among the students in the control group, even though they started off with similar social skills to those in the experimental group, basic social skills were in this case a relevant factor, as the students in the control group with low basic social skills showed a greater reduction between the pre-test and the post-test than those students in the group who had a high level of basic social skills. This would indicate that, when students are set to work in groups on their own, without the guarantee of any minimum conditions to ensure the practicing of social skills (Johnson and Johnson, 1991), we then run the risk of students not only not developing their social skills, but also that these social skills may in fact become worse, especially among those with greater difficulties to interact with their peers.

As for the students’ academic level, this seems to be a relevant factor for imparting information skills. Thus, just as with cooperative work, there seems to be a certain social benefit for those with a greater capacity (Tourón, 2012), the university students with the best academic results would obtain greater benefits in their imparting information skills when working cooperatively. The different cooperative techniques advocate equal participation, with sharing of responsibility, so that all the team members, within their own possibilities, can motivate, provide information, explain and assist the other members. In this sense, the academically most gifted students would be the ones who could offer the most help, which would explain why cooperative learning has a positive effect on these students in particular (Neber et al., 2001). In addition, our results in the control group indicate that, when working in a team without a teacher to provide a structure to guarantee minimum conditions, although a negative effect on the social skills of the team can be appreciated, a better academic performance would be a protective factor against the said effect. When there is no kind of control over the team members’ participation, some students can dominate interventions and contributions, while others contribute nothing. This can lead to negative attitudes toward teamwork that make the group dynamics more complicated (Mendo et al., 2017).

### Limitations

This research has various limitations, including: the exclusive use of self-reporting as the method for gathering data (such methods are not very robust against possible bias in responses introduced by the subjects themselves); the impossibility of greater control over the study variables in real situations in university classrooms; or the sample used makes the generalization of the results to students of other university degrees or educational levels more difficult. Also when assuming that the cause (cooperative learning) leads to the effect (social skills) it is important to keep in mind that there may be other confounding factors not explored in the studies (teaching and learning styles, student attitudes, previous experiences(…). The results should therefore be interpreted in the light of all these limitations.

## Conclusion

On the basis of the results set out here, and taking advantage of the educational paradigm of the EEES that approaches the teaching-learning process as cooperative work between teachers and students, as well as the growing importance of cooperation in all types of organizations (Eurofound, 2007; Gil and Alcover, 2008), we can conclude that the cooperative methodology is particularly relevant for the development of university students’ interpersonal, social, and teamwork competences; professional competences which will be decisive for their social and professional success.

The acquisition of the competences that define each university degree cannot be achieved through the exclusive use of traditional methods. From this perspective, we believe that cooperative learning is a valuable tool to generate some of the changes sought in the EHEA in the last decade, compatible with more traditional methods. In fact, students value positively the mix of traditional lecturing and cooperative learning tasks (Cavanagh, 2011). However, the application of cooperative learning in the university classroom is not without problems. The organizational structure, the competitive climate, and the emphasis on theoretical concepts for achieving academic success, do not favor its application (Darnon et al., 2009; Buchs et al., 2016).

It is important to stress that, in order to ensure the development of the said competences through cooperative learning, expert instruction is needed to make its use a constant in university spaces and guarantee the minimum conditions, with new quality spaces (teacher training adapted to new competencies, new competency evaluation tools, more practical classes, individual and group tutorial support, versatile classrooms; adequate student-teacher ratio, etc.), for the teams to work and practice their social relationship skills. The students are frequently asked to work with their colleagues on tasks to resolve problems, to explain and share their thought processes and to ask for help when confused; yet when the students have difficulties to communicate and resolve problems, learning can suffer. Giving them strategies and cooperative tools to use in these situations will help to ensure greater success in all future situations where cooperation and social interaction play an important role.

Lastly, it could be said that, although control over learning groups in the university context as far as the number of members in the groups, the basic social skills or the academic level is concerned, are all relevant; the development of the social skills necessary for working in teams depends, to a great extent, on the philosophy behind it. The differentiation and continuity of the methodology used by the teacher is what will make a real difference in their development.

## Author Contributions

All authors listed have made substantial, direct and intellectual contribution to the work, and approved it for publication. SM-L and BL-d-B analyzed and interpreted the data. SM-L drafted the work. SM-L, BL-d-B, M-IP-d-R, EF-C, and DI-G conceived and designed the work.

## Conflict of Interest Statement

The authors declare that the research was conducted in the absence of any commercial or financial relationships that could be construed as a potential conflict of interest.
